# Preparation of Chitin–Glucan Complex Aerogel from Mycelium Waste with Tunable Properties

**DOI:** 10.3390/gels12010041

**Published:** 2026-01-01

**Authors:** A. M. Abdel-Mohsen, Katerina Skotnicova, Rasha M. Abdel-Rahman, Josef Jancar

**Affiliations:** 1 Faculty of Materials Science and Technology, VSB—Technical University of Ostrava, 70800 Ostrava, Czech Republic; 2 Central European Institute of Technology, Brno University of Technology, Purkyova 656/123, 61200 Brno, Czech Republic; 3 Faculty of Chemistry, Materials Research Center, Brno University of Technology, Purkyova 464/118, 61200 Brno, Czech Republic

**Keywords:** chitin–glucan complex, *Aspergillus niger*, NaOH/urea solvent, aerogels, rheology, biocompatibility

## Abstract

Chitin–glucan complex (CGC) is a naturally occurring heteropolysaccharide in which chitin chains are covalently integrated with β-glucans, forming a rigid structural framework in fungal and yeast cell walls. CGC exhibits a broad spectrum of functional properties, including antimicrobial, antioxidant, adsorption, and tissue-regenerative activities; however, its technological exploitation has been severely constrained by its intrinsic insolubility in water and most common solvents. In this work, CGC was isolated from *Aspergillus niger* mycelial biomass and, for the first time, completely dissolved in a precooled aqueous NaOH/urea solvent system (12 wt.% NaOH, 8 wt.% urea) within 5 min at ambient temperature, yielding a clear and stable solution. The influence of alkali concentration on dissolution efficiency and solution stability was systematically examined. Structural integrity and covalent linkage between chitin/chitosan and glucan segments were confirmed using FTIR spectroscopy, two-dimensional NMR, and electron microscopy. The degree of deacetylation determined by NMR was approximately 25%. Rheological analysis revealed concentration- and temperature-dependent sol–gel transitions, with well-defined storage and loss moduli during gelation. Crosslinking with epichlorohydrin enabled the fabrication of lightweight, highly porous three-dimensional CGC aerogels. In vitro cytocompatibility studies using NIH 3T3 fibroblasts demonstrated no detectable cytotoxicity over 72 h. These results establish a green, efficient route for CGC dissolution and processing and highlight the promise of CGC aerogels as sustainable biomaterials for biomedical and environmental applications.

## 1. Introduction

The chitin–glucan complex (CGC) is a naturally occurring copolymer composed of chitin, a linear polysaccharide of *N*-acetyl-*D*-glucosamine, covalently linked to *β*-(1,3)- and *β*-(1,6)-glucans, which are glucose homopolymers [1,2,3]. CGC constitutes a major structural component of the fungal and yeast cell walls, where it plays a crucial role in maintaining mechanical integrity, rigidity, and resistance to environmental stress [4]. Due to its biodegradability, biocompatibility, and intrinsic bioactivity, CGC has emerged as a promising biomaterial for advanced applications [5]. Numerous studies have reported its antibacterial [3,6,7,8,9], antioxidant [10], and anticancer properties [11], as well as its use in cosmetic formulations [2], functional food additives, wound dressings [3,8], and therapeutic agents for metabolic disorders such as obesity and diabetes [12,13]. CGC is widely distributed among fungi and yeasts, including *Komagataella pastoris* [14], *Aspergillus niger* [9,14,15], *Schizophyllum commune* [3], *Gongronella butleri* [14,16], *Armillariella mellea* [17], *Pencellium* [18,19,20], and *Saccharomyces cerevisiae* [21].

Despite these valuable properties, the practical utilization of CGC in a wide range of applications remains limited by its extremely poor solubility in water and most conventional solvents [22,23]. Similarly to chitin [3,6,24,25], chitosan [3,7,8,9,26,27,28] and cellulose [29,30,31,32], CGC dissolves only in highly polar, often hazardous solvent systems such as dimethylacetamide—lithium chloride (DMAc/LiCl) [33], which are corrosive, toxic, and environmentally burdensome [12,34]. Additionally, the resulting solutions are frequently unstable and difficult to process, restricting their use in the fabrication of advanced materials. Poor solubility is primarily attributed to extensive intermolecular and intramolecular hydrogen bonding, together with strong covalent linkages between the glucan and chitin chains, imparting a highly ordered, crystalline structure resistant to disruption [8,35,36,37].

The chitin–glucan complex (CGC), derived from two fungal strains, *Komagataella pastoris* (CGCP) and *Aspergillus niger* (CGCKZ) (KiOnutrime-CGTM), was dissolved using ionic liquids. Films and gels were prepared with the ionic liquid; however, the effect of the ionic liquid on biological properties was not measured or evaluated [14]. Additionally, a porosity design of the mycelium chitin–glucan scaffold was developed via hydrothermal fabrication, and dual cross-linking was applied for wound healing applications. Chitin–glucan was extracted via microwave-based gluconic acid, this technique did not deacetylate the chitin chain in CGC, which is important in their applications [38].

To overcome these limitations, there is a growing demand for environmentally friendly, efficient, and scalable dissolution strategies that preserve the chemical integrity of CGC while enabling its processing into functional materials. Among the approaches explored, aqueous sodium hydroxide/urea systems have shown considerable promise for dissolving polysaccharides such as cellulose and chitosan under mild, low-temperature conditions, offering a nontoxic and cost-effective alternative to conventional solvents. However, to date, the application of this solvent system for the dissolution and regeneration of CGC has not been systematically investigated.

In recent years, aerogels derived from natural macromolecules have gained considerable research interest due to their combination of the unique structural features of conventional aerogels, such as high porosity, low density, and large surface area, with additional benefits of biocompatibility, biodegradability, non-toxicity, and environmental sustainability [39,40,41,42]. These advantages make them attractive candidates for a wide range of applications, including adsorption and separation processes for wastewater treatment. Various biopolymers, including chitosan, cellulose, gelatin, starch, and chitin, have been explored as precursors for aerogel fabrication, with chitin and its deacetylated derivative chitosan receiving particular attention due to their natural abundance, biocompatibility, and rich functional chemistry [41,43]. The presence of abundant amino (-NH_2_) and hydroxyl (-OH) groups in these polysaccharides allows strong interactions with pollutants, facilitating the simultaneous adsorption of heavy metal ions and organic dyes through electrostatic interactions, hydrogen bonding, and coordination mechanisms [44]. Despite these advantages, conventional chitin and cellulose-based aerogels often suffer from poor mechanical strength, which significantly limits their applicability in demanding environments such as continuous wastewater treatment systems [45,46,47].

To address this limitation, strategies such as chemical cross-linking have been employed to enhance structural integrity. Epichlorohydrin (ECH), for example, has been widely used as an effective cross-linker to improve the mechanical strength of hydrogels based on chitin and chitosan, suggesting its potential for strengthening the aerogel network [48,49,50]. In the present study, we explore a synergistic approach by utilizing chitin–glucan complex (CGC), a naturally occurring copolymer in the walls of fungal cells, as the precursor for aerogel fabrication. The combination of chitin and glucan components is expected to impart superior mechanical stability compared to single-component aerogels. Furthermore, chemical crosslinking with ECH was employed to reinforce the 3D network structure of the aerogels, thereby enhancing their mechanical robustness and broadening their potential applications. Building on our previous work, this study represents the first report on the fabrication of CGC-based aerogels from aqueous CGC solutions, providing a sustainable route for developing high-performance, renewable, and environmentally friendly functional materials.

The objective of this study was to extract, characterize, dissolve, and regenerate CGC aerogels using, for the first time, a sodium hydroxide/urea aqueous solvent system. CGC was isolated from the mycelial biomass of *Aspergillus niger* and successfully solubilized in a precooled NaOH/urea solution, producing a transparent aqueous solution. The effect of sodium hydroxide concentration on CGC solubility was systematically investigated, and the dynamic rheological behaviour of the polymer was analyzed as a function of concentration, temperature, and time. Following dissolution, the CGC solution was crosslinked with epichlorohydrin to fabricate three-dimensional (3D) aerogels. The regenerated aerogels were thoroughly characterized to evaluate their morphology, chemical structure, mechanical performance, and biological compatibility, highlighting their potential for biomedical and other advanced material applications.

## 2. Results and Discussion

### 2.1. Isolation and Dissolution of CGC

The isolation of CGC from native fungal mycelium was achieved through a sequential chemical treatment designed to remove noncovalently and covalently bound impurities. Initially, mycelium was treated with a low concentration of sodium hydroxide solution (1 wt.%, 5 h, 90 °C) to selectively remove surface-associated proteins and lipids by disrupting non-covalent interactions. Subsequently, 5 wt.% NaOH solution (90 °C, 6 h) was applied to cleave the covalent bonds between residual proteins, lipids, and the chitin–glucan matrix (CGC). This step was repeated four consecutive times to ensure complete removal of proteinaceous material, as confirmed by quantitative protein analysis, resulting in a purified CGC with negligible protein content.

### 2.2. Effect of NaOH Concentration

**Figure 1:**
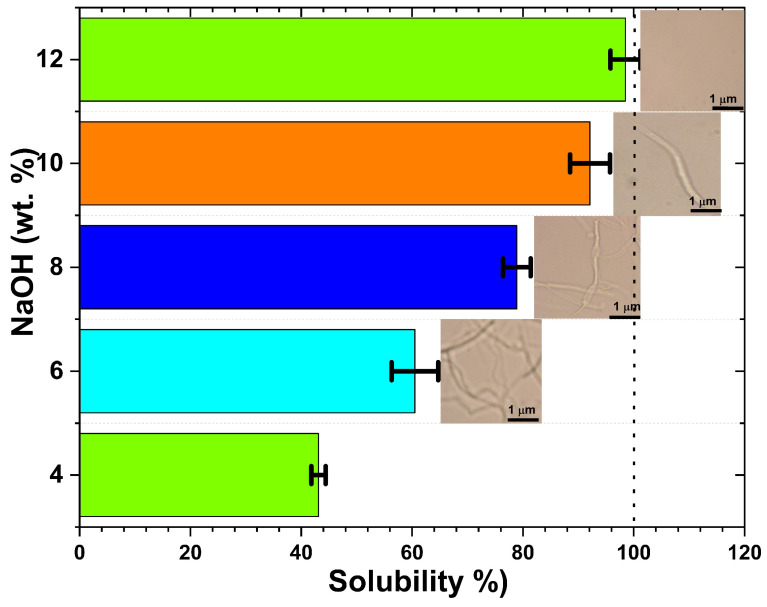
Effect of NaOH concentration on the solubility of chitin–glucan complex (CGC) at −15 °C. Insets show PLM images of CGC solutions after 5 min dissolution, illustrating the disappearance of crystalline domains with increasing alkali concentration.

The influence of sodium hydroxide concentration on the dissolution kinetics of the CGC was systematically investigated using aqueous NaOH solutions ranging from 4 to 12 wt.% (Figure 1). The solubility of 5 wt.% CGC increased markedly with NaOH concentration, reaching a maximum of 98% at 12 wt.% NaOH. This behaviour can be attributed to the concentration-dependent properties of sodium hydroxide/water hydrates. At low alkali concentrations (4, 6 wt.%), the hydrodynamic diameters of sodium hydroxide/water clusters may be too large to penetrate the crystalline domains of CGC, and insufficient alkali is available to disrupt the hydrogen-bonded network of chitin and glucan. In contrast, at NaOH concentrations greater than 8 wt.%, alkali molecules preferentially associate closely with polymer chains, potentially inducing the formation of Na/chitin/glucan ordered domains, analogously to the mercerization process, thus limiting solubilization. NaOH plays a pivotal role in CGC dissolution by penetrating both the amorphous regions and the crystallites, effectively disrupting the inter- and intramolecular hydrogen bonds between chitin and glucan chains (linear and branched).

The data obtained were consistent with previously published findings on the dissolution of chitin and cellulose using various aqueous solvent systems [35,51,52,53,54]. Polarized light microscopy (PLM) images (Figure 1) confirmed that dissolution in cold NaOH solution (–15 °C) occurs rapidly, in 5 min, and that solubility is strongly dependent on NaOH concentration. Transparent and homogeneous solutions were observed at intermediate alkali concentrations (8–12 wt.%), indicating efficient disruption of the CGC network.

The stirring speed rate has also been found to be of great importance in the preparation of solutions of higher CGC concentrations. CGC solubility increased with increasing stirring rates. It was found that a certain amount of chitin–glucan complex could be dissolved in the pre-cool solvent without any stirring. It indicated rapid dissolution of CGC in aqueous sodium hydroxide solution. However, the remaining CGC cannot be completely dissolved without stirring or under low-rate stirring. The increasing shear stress of high stirring rates is thought to help improve solvent diffusion and break off partially dissolved chains from the solid surfaces to accelerate dissolution.

### 2.3. Rheological Properties of GCG

**Figure 2:**
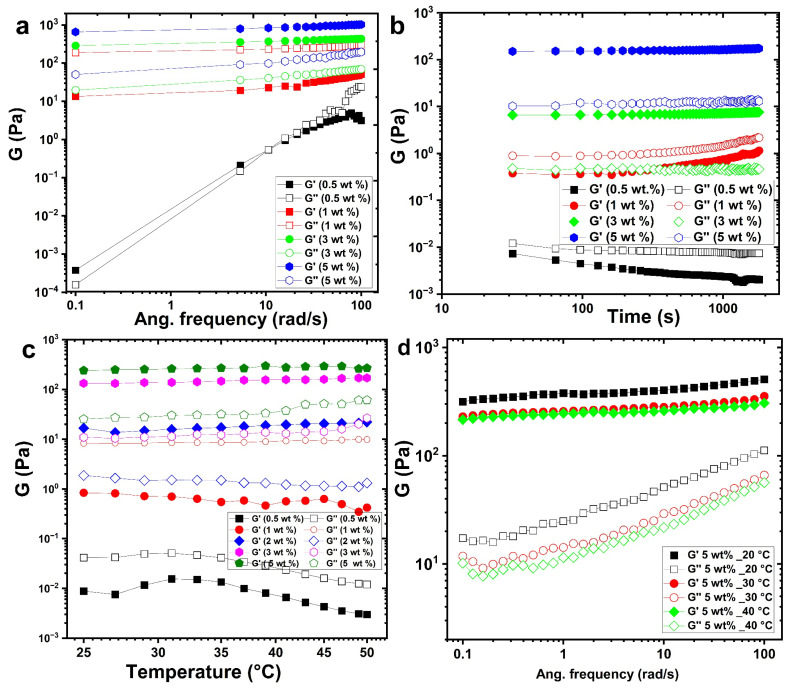
Rheological behavior of CGC solutions in NaOH/urea aqueous system: (**a**) frequency dependence of storage (G′) and loss (G″) moduli at 25 °C; (**b**) time evolution of G′ and G″ at different CGC concentrations; (**c**) temperature dependence of viscoelastic moduli during heating; (**d**) frequency-dependent moduli of 5 wt.% CGC at different temperatures.

Frequency sweep analysis serves as a fundamental method for probing the viscoelastic behaviour of dispersed polymeric systems, enabling classification into four canonical states: dilute solutions, entangled networks (concentrated solutions), weak gels, and strong gels. Figure 2a presents the frequency-dependent storage modulus (G′) and loss modulus (G″) of the solutions of the aqueous chitin–glucan complex (CGC) at 25 °C. At low concentrations of CGC (0.5–1 wt.%), G ‘dominantly’ dominates G′ across the entire frequency spectrum, indicative of a predominantly viscous response characteristic of dilute polymer solutions. However, both moduli increase with frequency, and the convergence of G′ and G″ at intermediate frequencies suggests the onset of polymer chain entanglements, marked by the crossover of the G′ and G″ curves. At higher concentrations (≥3 wt.%), G′ surpasses G″ at low frequencies (<10 s^−1^), reflecting a transition from liquid- to solid-like behavior consistent with weak gel formation. Temperature-dependent measurements show that at 25 °C, G′ and G′′ intersect near 0.1 s^−1^, indicating that the gelation temperature for 3 wt.% CGC lies just above this temperature. As the concentration increases from 3% by weight to 5% by weight, both moduli increase substantially and the separation between G ′ and G″ widens, evidencing the progressive dominance of elastic behavior (G′ ≫ G″) and a more robust gel network.

Time-dependent rheological studies (Figure 2b) reveal that the gelation dynamics is strongly concentration-dependent. Increasing the CGC concentration from 0.5 to 5% by weight at 25 °C leads to shorter gelation times and higher gel strengths. This effect is mainly attributed to chitin with a low degree of deacetylation, which promotes hydrophobic interactions at elevated temperatures. A higher chitin content enhances chain entanglements, reinforcing the gel strength. Interestingly, near the sol–gel transition (above 3 wt.%), ageing leads to a gradual decline in G′ and G″, indicating partial reversion from the gel state back to the sol state.

Temperature-dependent rheology (Figure 2c) demonstrates that at sub-gelation temperatures, G′ remains below G″, with both moduli decreasing at lower concentrations (0.5–1 wt.%). Upon heating to gelation temperature, G′ increases significantly than G″, and G′ exceeds G″ above the gelation threshold, signalling gel formation. Below the gelation point, the CGC solution remains transparent and stable, whereas a white-to-yellow gel forms at or above the gelation temperature. The frequency-dependent behaviour of a 5 wt.% CGC solution further illustrates the effect of gelation temperature on viscoelastic properties (Figure 2d). At 20 °C, well below the gelation temperature, G″ exceeds G′ at all frequencies, characteristic of a viscous liquid. On approach to gelation temperature, the difference between G′ and G″ diminishes, indicative of a weak gel exhibiting frequency-dependent moduli. At temperatures exceeding the gelation point (30–40 °C), G′ consistently exceeds G ′′ throughout the frequency range, and the elastic modulus becomes nearly frequency independent. These characteristics are typical of a strong gel, with G′ showing a plateau (slope ≈ 0) and G <′′ showing a minimum at intermediate frequencies. This behaviour is consistent with previous work in which CGC hydrogels were also developed [14,55,56].

**Figure 3:**
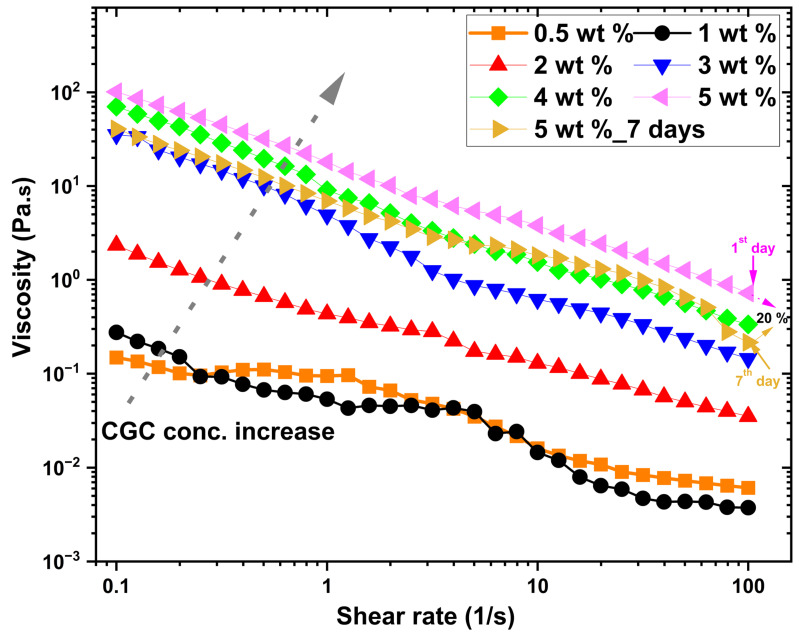
Steady shear viscosity as a function of shear rate for CGC solutions (0.5–5 wt.%) at 25 °C.

Figure 3 illustrates the variation in steady-state shear viscosity (Pa·s) as a function of shear rate for aqueous solutions of CGC at different concentrations ranging from 0.5 to 5 wt% at 25 °C. At lower concentrations (c < 2 wt.%), the viscosity/shear rate relationship appears nearly linear, indicating Newtonian-like behaviour, where viscosity remains constant regardless of the applied shear rate in the low shear regime. This suggests minimal intermolecular interactions and low structural complexity within the solution at these dilute conditions. As the CGC concentration increases, the overall viscosity of the system increases, which can be attributed to enhanced molecular interactions and the formation of larger polymeric aggregates. At higher concentrations, the viscosity/shear rate profiles exhibit a clear nonlinear decline at elevated shear rates, which is characteristic of shear-thinning or pseudoplastic behaviour. This phenomenon is commonly observed in biopolymer solutions such as chitosan [57,58,59], hyaluronan [60,61,62] and carboxymethyl cellulose [63,64,65], where polymer chain alignment and disentanglement occur in response to applied shear, leading to a reduction in flow resistance. Furthermore, this shear-dependent behaviour indicates that the applied mechanical force induces a preferential orientation of the CGC macromolecules along the flow direction, disrupting intermolecular entanglements and transient networks, which in turn reduces the effective viscosity. A comparison between freshly prepared CGC solutions (5 wt.%) and those stored for 7 days at 25 °C reveals a noticeable decrease in viscosity of approximately 20%, highlighting potential structural degradation or rearrangement over time, possibly due to partial hydrolysis, molecular disentanglement, or microbial activity.

**Figure 4:**
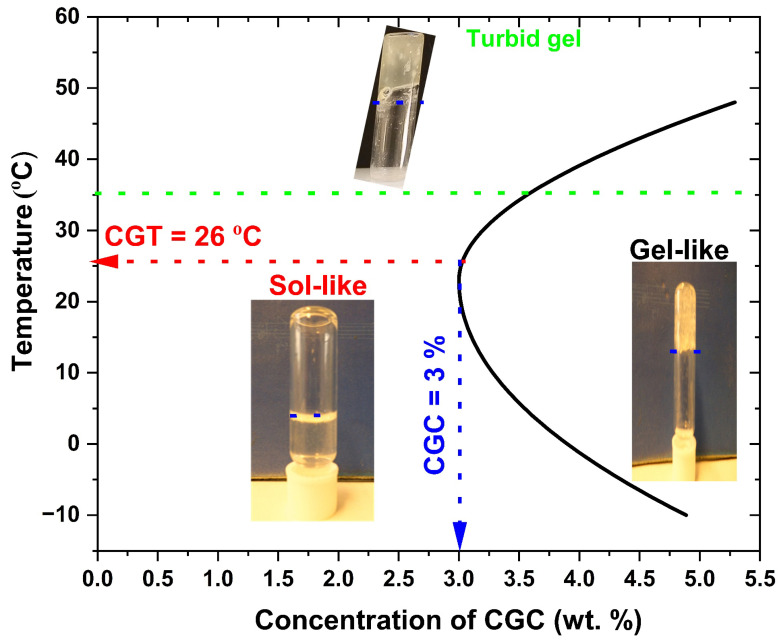
Sol–gel phase transition diagram of CGC solutions at different concentrations determined by the test tube inverting method.

The test tube inverting method (TTIM) enabled direct visualization of sol–gel transitions and allowed determination of the critical gelation temperature (CGT), defined as the lowest temperature at which gelation occurs, and the critical gelation concentration (CGC), the minimum concentration required for gel formation. Figure 4 shows that the sol–gel transition behaviour of CGC is strongly dependent on CGC concentration. At temperatures below (−10 °C), all tested concentrations dissolved in aqueous sodium hydroxide exhibited liquid-like properties with low viscosity, indicating favorable injectability. In this study, CGT and CGC were identified as 26 °C and 3 wt.%, respectively. At 3 wt.% concentration, the first sol-to-gel (SG) transition was observed at 26 °C, forming a translucent gel that persisted until 30 °C, at which point it became an opaque white gel (Figure 4). A second phase transition, corresponding to a gel-to-suspension (GS) shift, occurred at 40 °C, indicating stronger intermolecular interactions between CGC functional groups and the development of a more rigid network structure.

### 2.4. Chemical Structure of CGC

**Figure 5:**
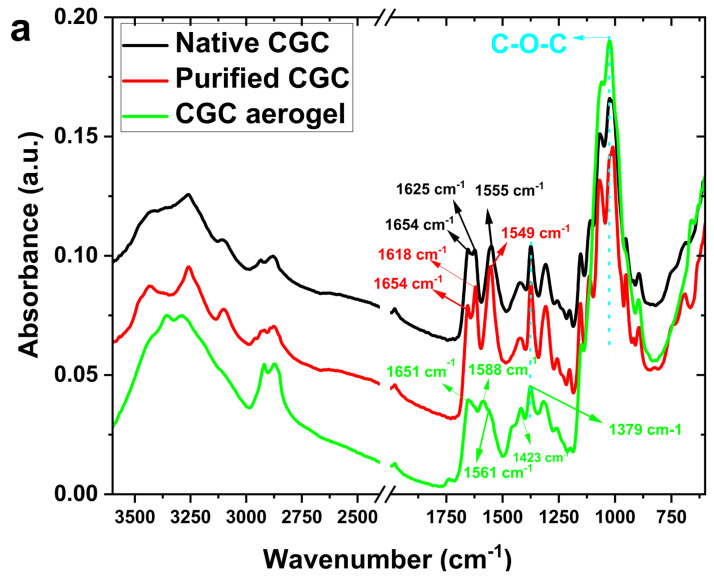

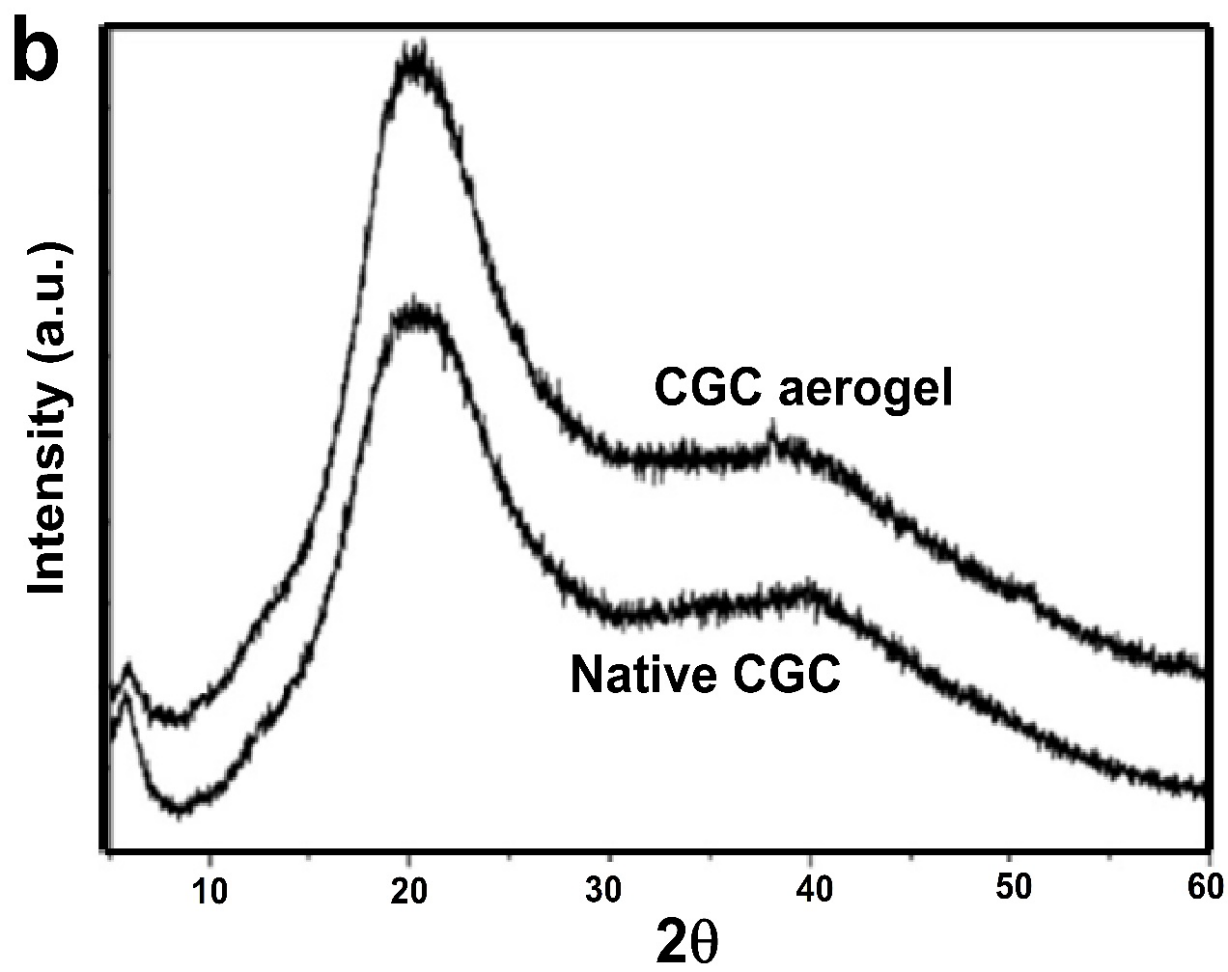
(**a**) FTIR spectra of native CGC and CGC aerogels; (**b**) XRD patterns of native CGC and regenerated CGC aerogels.

The chemical structure of CGC aerogels was characterized through Fourier Transform Infrared (FTIR) spectroscopy, as illustrated in Figure 5a. The FTIR spectrum of native CGC exhibits prominent absorption bands near 3400 cm^−1^ and 1340 cm^−1^, corresponding to the stretching vibrations of hydroxyl (O–H) and C–N groups, respectively [3,6,10]. Additionally, characteristic bands observed at 890 and 1375 cm^−1^ confirm the presence of *β*-glycosidic linkages within the polysaccharide backbone [66,67]. The absorption band at approximately 1654 cm^−1^ is attributed to the C=O stretching vibration (amide I) of the acetyl groups present in chitin. An increase in glutaraldehyde (GLA) concentration introduces a new band around 1555 cm^−1^, indicative of N–H bending vibrations (amide II) associated with amine functionalities. Further distinct bands at 1150 cm^−1^ and 1040 cm^−1^ correspond to the stretching vibrations of C–O–C and C–O bonds, respectively, suggesting that epichlorohydrin (ECH) reacts with the hydroxyl groups of CGC to form covalent linkages. This reaction involves the opening of the ECH epoxide ring and the subsequent release of a chlorine atom [46,68]. The absence of epoxide-related absorption peaks, specifically the weak ring-stretching band (1280–1230 cm^−1^), the strong asymmetric ring deformation band (950–815 cm^−1^), and the strong symmetric ring deformation band (880–750 cm^−1^) confirms the completion of the ECH crosslinking reaction and the effective elimination of residual ECH after neutralization and freeze-drying. Furthermore, the weakened peak observed at 1028 cm^−1^ in Figure 5a can be attributed to the deformation in the chitin–glucan matrix, providing evidence of the formation of the ether bond through the etherification reaction between ECH and CGC (Figure 7b).

The crystallographic characteristics of native CGC and the CGC aerogel (5 wt.%) were examined using X-ray diffraction (XRD), as presented in Figure 5b. The XRD pattern of native CGC exhibited two distinct diffraction peaks at 2θ = 5.8° and 20.4°, corresponding to the crystallographic planes (020) and (110), respectively, indicating a high degree of structural order and crystallinity. On the contrary, the CGC aerogel showed broader and less intense peaks at approximately 2θ = 5.4° and 20.2°, suggesting partial reduction in crystallinity associated with the aerogel formation process [69,70,71]. Nevertheless, the preservation of these characteristic peaks confirms that the dissolution and fabrication steps did not significantly alter the fundamental crystalline framework of the CGC. Moreover, the absence of any residual diffraction peaks attributable to urea or sodium hydroxide in the CGC aerogel pattern indicates the successful removal of these reagents during processing. This observation further supports the occurrence of a carbamate interaction between the CGC and the NaOH/urea system during dissolution, followed by the complete elimination of unreacted species upon gel formation and drying.

**Figure 6:**
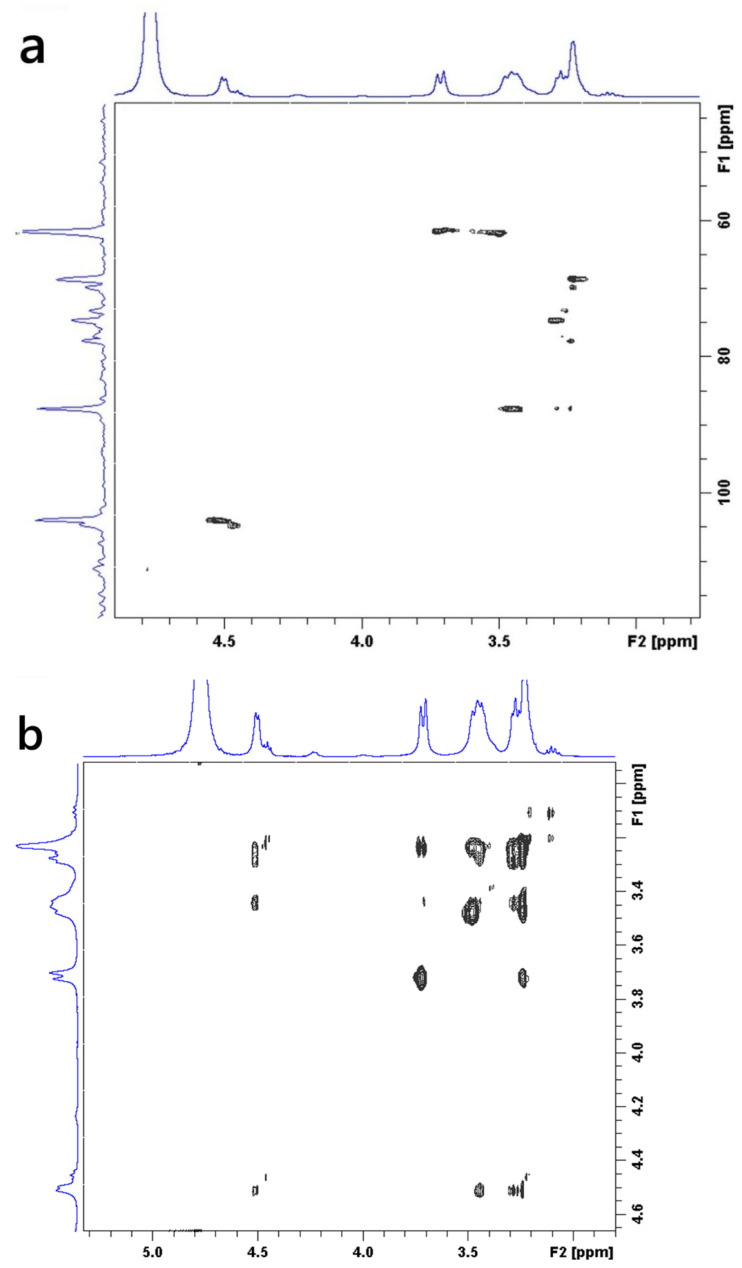
Two-dimensional NMR spectra of CGC dissolved in NaOH/urea/D_2_O: (**a**) ^1^H–^13^C HSQC spectrum; (**b**) ^1^H–^1^H COSY spectrum.

The structural architecture of the CGC was elucidated using ^1^H-NMR (Figure S1) and two-dimensional heteronuclear (^13^C–^1^H) HSQC (Figure 6a) and homonuclear (^1^H–^1^H) COSY (Figure 6b) NMR spectroscopy. Figure S1 shows the ^1^H-NMR spectrum of the CGC in urea/sodium hydroxide/D_2_O. Anomeric proton signals at 4.2 and 4.9 ppm correspond to (13)/(16) glucan units, while a resonance at 2.2 ppm is attributed to *N*-acetyl protons of the chitin fraction. The signal at 4.7 ppm confirms a *β*-glycosidic configuration [7,66,71]. From the NMR data, the degree of deacetylation (DDA) of CGC was approximately 25%. Two-dimensional heteronuclear (^13^C–^1^H) HSQC (Figure 6a) and homonuclear (^1^H–^1^H) COSY (Figure 6b) NMR spectroscopy enabled the precise assignment of all ^1^H and ^13^C resonances observed in CGC and the corresponding reference compounds, based on different carbon-hydrogen (C/H) and proton-proton (H/H) cross-peaks. The two-dimensional NMR spectra of CGC, dissolved in a urea/NaOH/D_2_O solvent system, clearly confirmed the *β*-configuration of D-glucosyl residues, as evidenced by the characteristic anomeric signal at *δ* 103.64 ppm. The resonance at *δ* 61.43 ppm was assigned to the C-6 carbon of the branched (1→3,6)-*β*-D-glucosyl residues. The downfield shift in this substituted C-6, relative to the unmodified C-6 of standard methyl glycosides, is attributed to the *β*-effect of glycosylation. Furthermore, the signal at *δ* 87.37 ppm corresponds to C-3 atoms of (1→3)-linked *β*-D-glucosyl and (1→3,6)-*β*-D-glucosyl residues, whose downfield displacement also reflects glycosidic substitution effects [72].

Comprehensive interpretation of the 2D 1H/^13^C NMR spectra (Figures S1 and Figure 6a,b) indicates that CGC is composed of covalent links involving *β*-(1→4) glycosidic bonds within the linear glucosamine/*N*-acetylglucosamine sequences of the chitin/chitosan backbone, *β*-(1→4) connections between the chitin, chitosan and glucan chains, and *β*-(1→3,1→6) glucosidic residues that show branching at the C-6 position of one of the units linked (1–3) link. These spectral characteristics collectively confirm the hybrid polysaccharide structure and the presence of interlinked chitin–glucan domains within the CGC framework.

**Figure 7:**
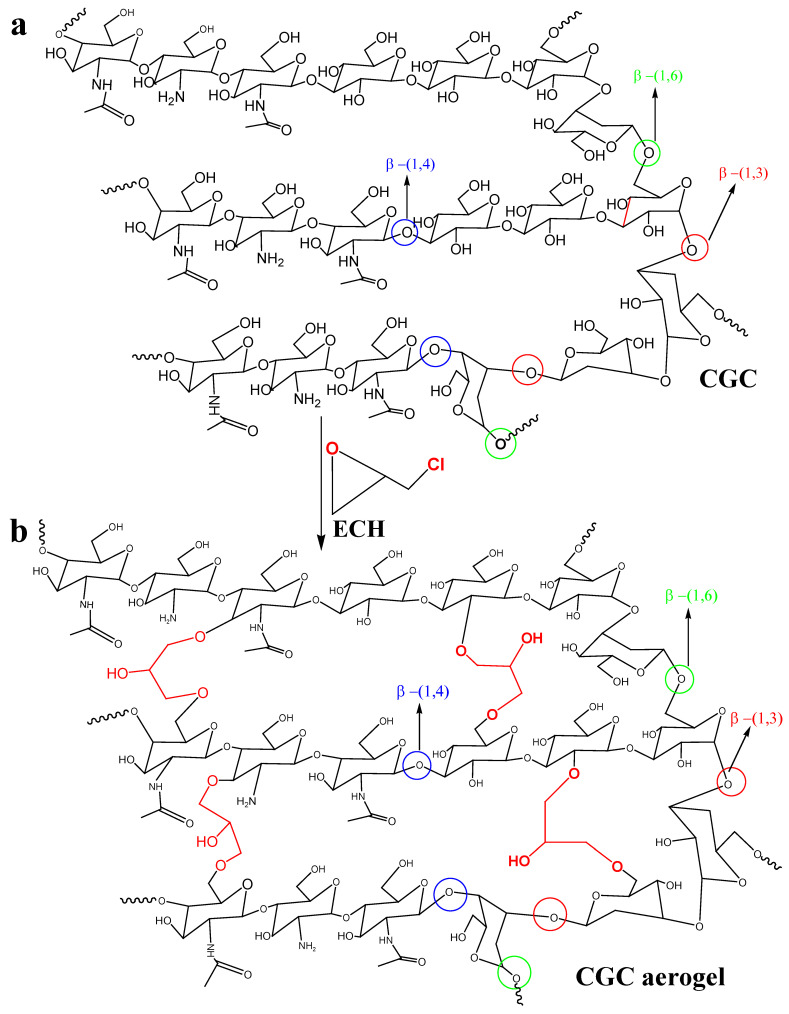
Schematic representation of the proposed molecular organization of chitin–glucan complex in *Aspergillus niger* cell walls (**a**) and chemical crosslinking mechanism of CGC aerogels (**b**).

^1^H-^13^C heteronuclear correlation of CGC in NaOH/D_2_O (a): TOSCY of CGC in NaOH/D_2_O (b). From the data of FT-IR and two-dimensional NMR spectra, presented in our work, a tentative scheme of the organization of glucan of the and covalently associated polymers found in the cell wall of *Aspergillus niger* can be presented in Figure 7a. The *β*-1, 3-Glucan is the main component of the alkali-insoluble fraction of *Aspergillus niger* cell wall, and it is highly branched with *β*-1, 6 links that make up a three-dimensional network without reducing ends in chitosan and glucan parts [73]. Chitin and *β-glucan were covalently anchored to these non-reducing* ends, producing a large heteropolymer complex. Chemically crosslinked chitin–glucan complex aerogels (CGC) were successfully prepared using a facile process that involved mixing and freeze drying (as shown in Figure 7b). No additional initiators or chemicals were required. Based on Figure 5 and Figure 6, our current hypothesis of the chronological events in the biosynthesis of the Aspergillus niger polysaccharide network is as follows: biosynthesis of *Aspergillus niger*. The *β*-glucan ore of *Aspergillus niger* is branched via *β*-1, 6 linkages. The *β*-1,3/*β*-1,6-glucan fraction of -1,3/-1,6-glucan represents 85% of the total glucan of the cell wall and contains 3% of branch points [74] andthe fraction is a complex structure associating chitin to *β*-1,3-glucan via a *β*-1,4-linkage [75,76]. This cross-linking is essential for the alkali insolubility of *β*-glucan in yeast, as well as filamentous fungi [77].

**Figure 8:**
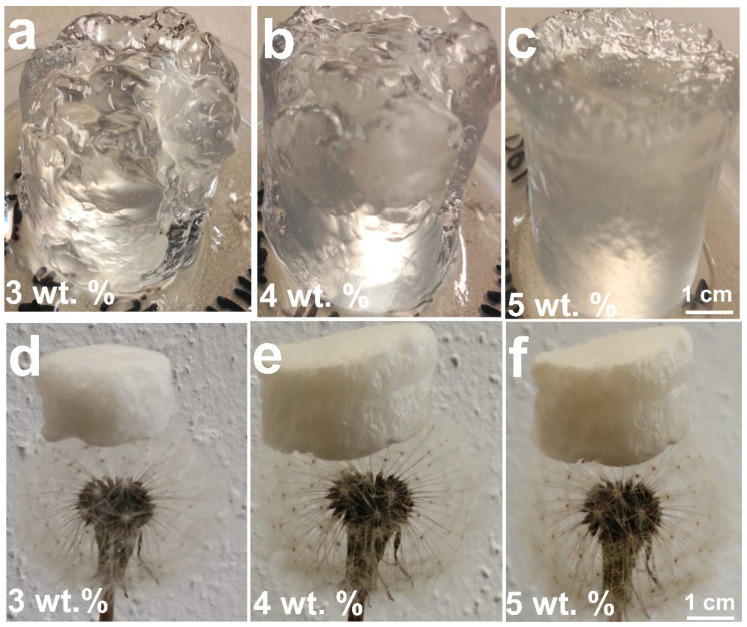
Photographs of CGC hydrogels and aerogels prepared at different concentrations: hydrogels at (**a**) 3 wt.%, (**b**) 4 wt.%, and (**c**) 5 wt.%; corresponding freeze-dried aerogels at (**d**) 3 wt.%, (**e**) 4 wt.%, and (**f**) 5 wt.%.

Figure 8 illustrates the morphological transformation of CGC aerogels at various stages of fabrication. Transparent CGC hydrogels (3–5 wt.%) were obtained following crosslinking with epichlorohydrin (ECH) and subsequent neutralization with deionized water. On the contrary, samples with lower concentrations of CGC (1–2 wt.%) failed to form stable three-dimensional aerogels, indicating insufficient network integrity at low polymer content. At moderate concentrations (approximately 3 wt.%), a transparent hydrogel was successfully produced (Figure 8a) with a high swelling capacity of nearly 3300%. Increasing the CGC concentration to 4 and 5 wt.% (Figure 8b,c) resulted in semitransparent hydrogels that exhibited reduced swelling ratios of approximately 2500% and 2100%, respectively. This decrease in swelling behaviour can be attributed to enhanced interactions between ECH and functional groups within the CGC matrix, leading to denser crosslinking. The chemically crosslinked CGC hydrogels synthesized through the combined sol–gel and thermal treatment processes exhibited high optical transparency and minimal volumetric shrinkage. The volumetric similarity between the hydrogels obtained after neutralization and the corresponding freeze-dried aerogels (Figure 8d–f) indicates structural stability throughout the drying process. Compared to low-concentration samples, the etherification reaction between ECH and CGC established not only covalent crosslinks among chitin and glucan chains but also regenerated hydroxyl groups consumed during crosslinking. This process promoted additional intermolecular hydrogen bonding and physical entanglement between polymer chains, thereby enhancing the overall network density and structural integrity of the resulting aerogels.

### 2.5. SEM of CGC Aerogels

**Figure 9:**
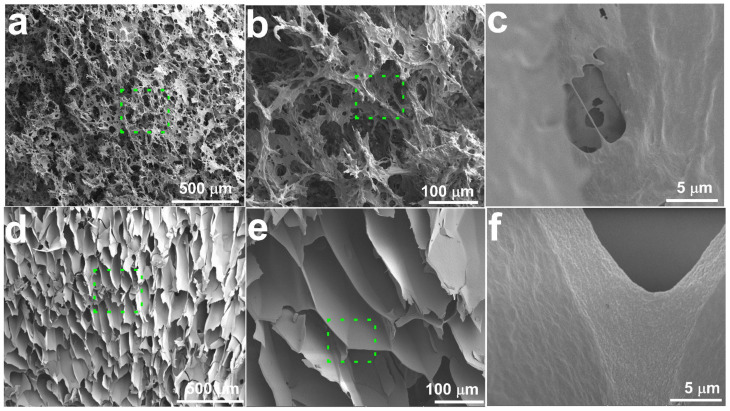
SEM images of (**a**–**c**) native CGC and (**d**–**f**) CGC aerogel prepared from 5 wt.% CGC, showing interconnected macroporous architecture.

The morphology of the isolated chitosan sample was studied using a scanning electron microscope. SEM images with different magnifications (Figure 9a–c) of native CGC. It was observed that the CGC surface shows a fibrillar structure with a rough surface after demineralization and deproteinization treatments. The morphologies of the obtained CGC aerogels of 5 wt% CGC concentrations were observed by SEM and shown in Figure 9d–f. Obviously, a well-defined, interconnected, three-dimensional porous structure was formed when the CGC chains were regenerated from NaOH/urea aqueous solution and chemically crosslinked using ECH (5%), exhibiting a different hierarchical porous structure. When the CGC concentration was 5 wt%, the aerogel showed hierarchical porous structure with a pore size in the range of 50–75 μm, and its three-dimensional network structure was constructed using a filamentary CGC matrix (Figure 9d). When the CGC concentration was 5 wt.%, the aerogel showed a hierarchical porous structure with a pore size in the range of 50–75 μm, and its three-dimensional network structure was constructed using a filamentary CGC matrix (Figure 9d). From the cross section of the CGC aerogels (Figure 9e,f), the layer was compacted with diameters of approximately 2 μm. This indicated that the semi-rigid molecular chain played an important role in supporting the pore wall [78].

### 2.6. Cell Viability Assay of CGC Aerogel

**Figure 10:**
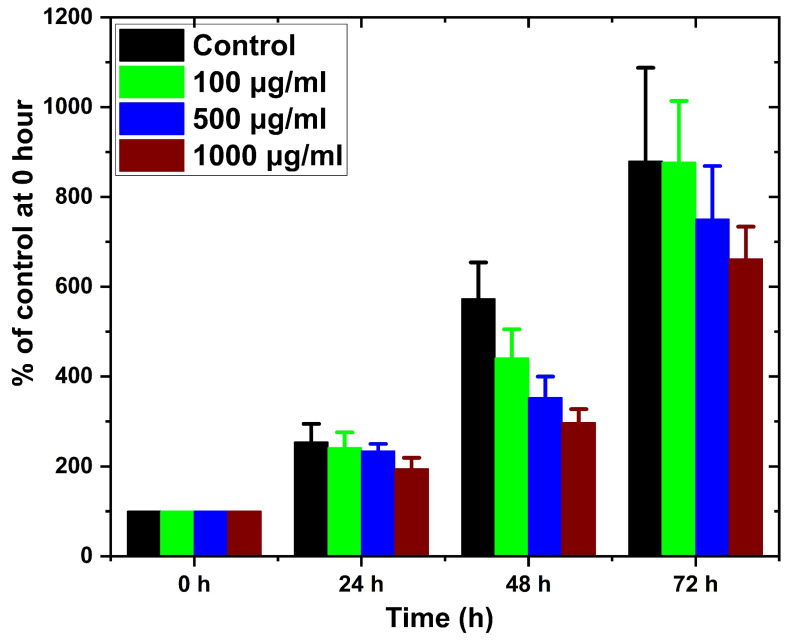
Cell viability of NIH-3T3 fibroblasts after exposure to CGC aerogels at different concentrations for 24, 48, and 72 h.

Figure 10 presents the effects of CGC aerogels on the viability of *NIH-3T3* fibroblasts exposed to concentrations ranging from 100 to 1000 µg/mL for up to 72 h. The results demonstrated that cell proliferation and metabolic activity remained consistent with those of the untreated control group, indicating that the CGC aerogels did not exert cytotoxic effects in the entire concentration (100 to 1000 µg/mL) and in the time range tested (24, 48, and 72 h). These findings confirm that sodium hydroxide/urea, employed as a green solvent for the dissolution of CGC and 3D aerogel fabrication, does not compromise cell compatibility. Consequently, CGC aerogels exhibit promising potential for biomedical applications, particularly in controlled drug delivery and bone tissue regeneration strategies.

## 3. Conclusions

Three-dimensional biobased aerogels were successfully prepared from a chitin–glucan complex (CGC) obtained from *Aspergillus niger*, constituting the first demonstration of aerogel fabrication using this biomass source. The process involved dissolution of CGC in a precooled aqueous NaOH/urea system, followed by freeze-drying to produce a highly porous structure. Structural characterization by FTIR and two-dimensional NMR spectroscopy verified a chitin backbone covalently linked to glucan side chains, with the glucan fraction primarily composed of low-branched units. CGC solutions exhibited a sol–gel transition at a concentration of 3 wt.% and a gelation temperature near 26 °C, indicating clear dependence on both temperature and polymer concentration. In vitro cytocompatibility assays showed no detectable cytotoxic effects on NIH-3T3 fibroblasts after three days of exposure to CGC concentrations between 100 and 1000 µg mL^−1^. Overall, the findings demonstrate that CGC at 5 wt.% can be efficiently dissolved in precooled NaOH/urea and processed into aerogels with adjustable structural characteristics. These CGC-based three-dimensional aerogels represent promising candidates for biofunctional scaffold applications, including controlled drug delivery systems and skin tissue engineering.

## 4. Materials and Methods

The CGC was extracted from the mycelial biomass of *Aspergillus niger* (AG), cultivated under controlled conditions as the primary biological source. Analytical grade sodium hydroxide (NaOH), acetic acid (CH_3_COOH), ethanol (C_2_H_5_OH), hydrochloric acid (HCl), epichlorohydrin (ECH) and isopropyl alcohol (IPA) were obtained from Penta Chemicals, Prague, Czech Republic and used without further purification. Ultrapure Milli-Q water was utilized throughout all preparation, washing, and analytical procedures to ensure consistency and eliminate potential contaminants.

### 4.1. Isolation of CGC

The dry mycelium obtained from *Aspergillus niger* (AG) was subjected to a 24 h alkaline treatment with 5% sodium hydroxide at 90 °C to remove surface-bound proteins and lipids. After this treatment, the resulting mycelial residue was separated by centrifugation and thoroughly washed with Milli-Q water until the washing reached a pH of approximately 7.5. The mycelium was then treated sequentially with 75% isopropyl alcohol (IPA) and absolute IPA to remove any remaining organic solvents and impurities. The purified mycelial cake was subsequently dried at 50 °C for 24 h to produce the final CGC product.

### 4.2. Dissolution and Preparation of CGC Aerogels

The chitin–glucan complex with a defined degree of deacetylation (DDA) was used as the raw biopolymeric material in all experiments. CGC solutions were prepared at different concentrations by dispersing CGC in mass fractions ranging from 1 to 5 wt.% into aqueous sodium hydroxide (NaOH) solutions of varying concentrations (5–12 wt.%). The dissolution was facilitated under subambient conditions. Specifically, a measured amount of CGC was added to NaOH of the desired concentration and stirred vigorously for 10 min at room temperature (−25 °C) to ensure uniform dispersion. The mixture was then subjected to a freeze–thaw protocol, consisting of freezing at −15 °C followed by thawing at ambient temperature. This cycle was repeated three times, which disrupted the native hydrogen bonding and crystalline domains within the CGC network, thereby enhancing the solubilization and resulting in a transparent CGC solution. further improve the stability of CGC under alkaline conditions, urea (8 wt.%) was introduced into the solutions after dissolution.

The solubilized CGC was then cross-linked using epichlorohydrin (ECH). The crosslinker with a concentration of 5% was added dropwise to the CGC solution while maintained at 0 °C and the reaction mixture was stirred continuously for 1 h under the same temperature to ensure homogeneous crosslinking. Before drying, the CGC/ECH mixture was centrifuged at 7500 rpm for 30 min at 0 °C to eliminate entrapped air bubbles and residual particulates. The resulting material was then subjected to thermal drying in a convection oven at 60 °C for 5 h.

### 4.3. Characterization of CGC

The structural, thermal, spectroscopic, rheological, and morphological properties of CGC were investigated systematically. X-ray diffraction (XRD) (Bruker D8 Advance, Karlsruhe, Germany) was used to analyze crystallinity under standard operating conditions (40 kV, 30 mA, 2θ = 5–60°). ATR-FTIR spectroscopy (Bruker Optik GmbH, Ettlingen, Germany) was used to identify functional groups within 4000–600 cm^−1^. For nuclear magnetic resonance (NMR) spectroscopy, CGC dissolved in NaOH/urea/D_2_O was analysed on a Bruker Avance 500 MHz system (Karlsruhe, Germany), with TOCSY, HSQC and DOSY spectra acquired to resolve molecular structure and dynamics. Rheological measurements (ARES rheometer, TA Instruments, New Castle, IN, USA) were performed on CGC dispersions (0.5–5 wt.%) using steady shear and oscillatory tests to determine viscosity, storage (G′) and loss (G″) moduli, including gelation temperature behaviour. The sol–gel transition of CGC solutions was evaluated using the test tube inverting method (TTIM) under a controlled temperature ramp from −10 to 50 °C at a heating rate of 1 °C/min in a water bath. The true CGC solutions of defined concentrations were prepared by dissolving the polymer in 1 mL of cold aqueous sodium hydroxide solution, followed by stirring in an ice-cooled bath (10 °C) until complete dissolution was achieved. For gelation assessment, aliquots of the solutions were transferred to glass vials and equilibrated at specific temperatures for 15 min. The samples were then inverted and the absence of flow within 30 s was considered evidence of gel formation. The sol–gel transition temperature was defined as the point at which the polymer system shifted from a flowable (sol) state to a nonflowable (gel) state upon sequential temperature increases of 1 °C.

The dissolution of the chitin–glucan complex (CGC) was observed by the disappearance of the highly crystalline short fibrils using a polarized light microscope (PLM, Olympus Axiovert 200M, Tokyo, Japan). The percentage of solubility (%) was determined by characterizing the amount of chitin–glucan complex (CGC) dissolved and expressed as S (%) = [S_1_/(S_1_ + S_2_)] × 100, where S1 was the weight of the dissolved CGC and S2 was the combined weight of gel and insoluble CGC separated by centrifugation and then dry process. Scanning electron microscopy (SEM) was performed on cryofractured, gold-coated scaffolds to examine microstructural morphology and quantify the pore size distribution. Cell Viability Assay: *NIH 3T3* mouse fibroblasts (Sigma-Aldrich, passages 10–20, Brno, Czech Republic) were cultured in DMEM supplemented with 10% FBS, 0.3 mg/mL L-glutamine, 100 U/mL penicillin, and 0.1 mg/mL streptomycin at 37 °C at 7.5% CO_2_. CGC aerogels (5 wt.%) were neutralized to pH 7.4 with Milli-Q water (Prague, Czech Republic), autoclaved at 121 °C for 20 min, and equilibrated in culture medium at 1000 µg/mL. Cells were seeded at 3 × 10^3^ per well in 96-well plates and allowed to adhere for 24 h before treatment with aerogel suspensions (100–1000 µg/mL). Cell viability was assessed at 0, 24, 48, and 72 h using the MTT assay. Briefly, MTT solution was added and incubated for 2.5 h at 37 °C. After the supernatant, cells were lysed for 30 min with gentle shaking to solubilize formazan crystals. Absorbance was measured at 570 nm using a VersaMax microplate reader (Molecular Devices, San Jose, CA, USA) in triplicate.

## Data Availability

Data will be made available on request.

## Supplementary Materials

The following supporting information can be downloaded at: https://www.mdpi.com/article/10.3390/gels12010041/s1, Figure S1. ^1^H-NMR of the CGC after dissolution in urea/sodium hydroxide/D_2_O solution.
