# Supplementary material for: Preparation of Chitin–Glucan Complex Aerogel from Mycelium Waste with Tunable Properties
Source: Gels. 2026 Jan 1;12(1):41. doi: 10.3390/gels12010041 (PMC12840671; doi:10.3390/gels12010041)
Supplement: Supplementary file 1 [file gels-12-00041-s001.zip › gels-4026045-supplementary.pdf]

Supporting information

Preparation of Chitin–Glucan Complex Aerogel from Mycelium Waste with Tunable Properties

A. M. Abdel-Mohsen [1,2,*], Katerina. Skotnicova [1], Rasha. M. Abdel-Rahman [2] and Josef. Jancar [2,3]

[1]Faculty of Materials Science and Technology, VSB—Technical University of Ostrava, 70800 Ostrava, Czech Republic; katerina@vsb.cz

[2]Central European Institute of Technology, Brno University of Technology, Purkyova 656/123, 61200 Brno, Czech Republic; rasha.abdelrahman@ceitec.vutbr.cz (R.M.A.-R.); jancar@ceitec.vutbr.cz (J.J.)

[3]Faculty of Chemistry, Materials Research Center, Brno University of Technology, Purkyova 464/118, 612 00 Brno, Czech Republic

* Correspondence: abdelmohsen.moustafa.abdellatif@vsb.cz

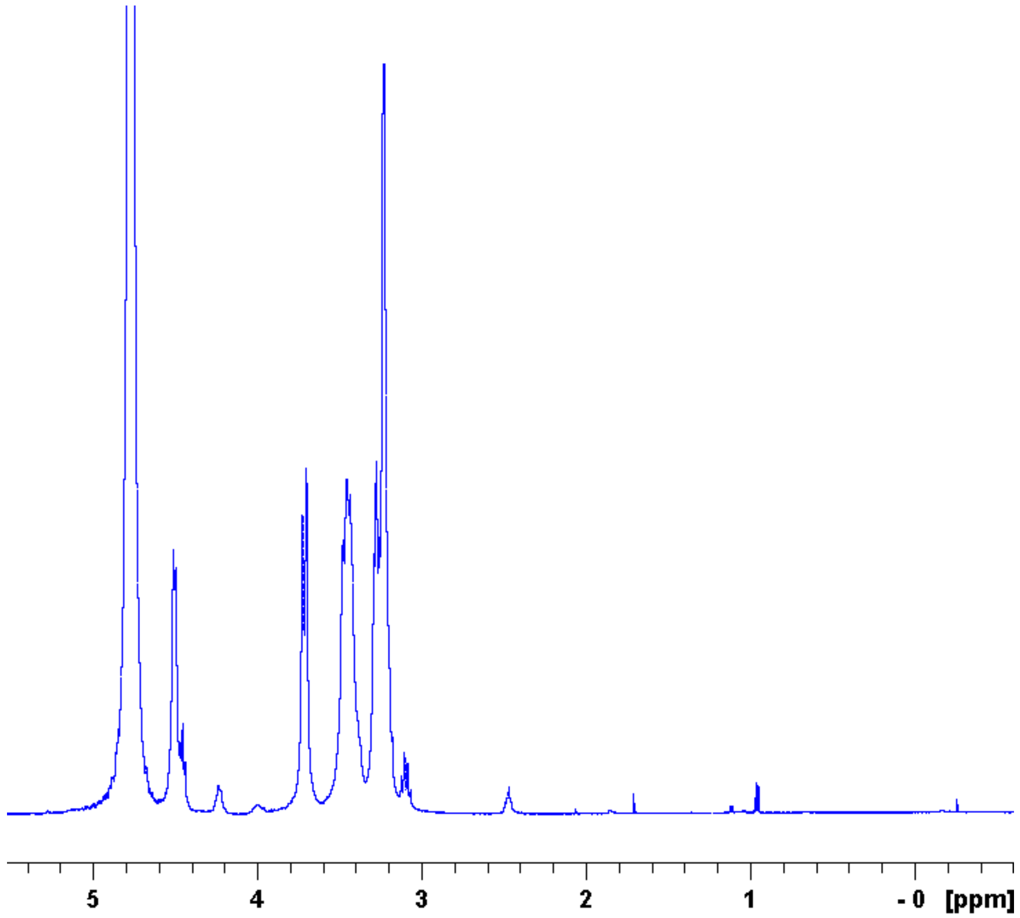

Figure S1. ^{1}H-NMR of the CGC after dissolution in urea/sodium hydroxide/D_2O solution
